# Effects of salinity on the growth, physiology and relevant gene expression of an annual halophyte grown from heteromorphic seeds

**DOI:** 10.1093/aobpla/plv112

**Published:** 2015-09-18

**Authors:** Jing Cao, Xiu Yun Lv, Ling Chen, Jia Jia Xing, Hai Yan Lan

**Affiliations:** Xinjiang Key Laboratory of Biological Resources and Genetic Engineering, College of Life Science and Technology, Xinjiang University, Urumqi 830046, China

**Keywords:** Antioxidative system, descendants from dimorphic seeds, gene expression, physiological response, salinity tolerance, *Suaeda aralocaspica*

## Abstract

Seed heteromorphism provides plants with alternative strategies for survival in unfavourable environments. However, the response of descendants from heteromorphic seeds to stress has not been well documented. *Suaeda aralocaspica* is a typical annual halophyte, which produces heteromorphic seeds with disparate forms and different germination characteristics. To gain an understanding of the salt tolerance of descendants and the impact of seed heteromorphism on progeny of this species, we performed a series of experiments to investigate the plant growth and physiological parameters (e.g. osmolytes, oxidative/antioxidative agents and enzymes), as well as expression patterns of corresponding genes. Results showed that osmolytes (proline and glycinebetaine) were significantly increased and that excess reactive oxygen species (${{\text{O}}_{2}}^{-},$ H_2_O_2_) produced under high salinity were scavenged by increased levels of antioxidant enzymes (superoxide dismutase, ascorbate peroxidase and glutathione reductase) and corresponding antioxidants (ascorbic acid and glutathione). Moreover, enhancement of phosphoenolpyruvate carboxylase activity at high salt intensity had a positive effect on photosynthesis. The descendants from heteromorphic seeds presented no significant difference in performance with or without salinity. In conclusion, we found that high salinity induced the same active physiological responses in plants from heteromorphic seeds of *S. aralocaspica*, there was no carry-over of seed heteromorphism to plants: all the descendants required salinity for optimal growth and adaptation to their natural habitat.

## Abstract (precis)

Seed heteromorphism provides plants with alternative strategies for survival in unfavourable environments, which may not only increase the chances of successful germination, but may also have an impact on characters of the descendants. However, Cao *et al*. found that the different properties of the dimorphic seeds of *Suaeda aralocaspica* (i.e. black and brown) had no effects on their descendants' growth and physiological responses to salinity: all descendants required salinity for optimal growth and adaptation to their natural habitat.

## Introduction

Seed heteromorphism is a phenomenon in which an individual plant is able to produce different types of seeds with diverse morphologies or germination and dormancy behaviours (Sorensen 1978; Mandák and Pyšek 2001; Brändel 2004, 2007). These adaptations evolved in response to adverse environments (Imbert 2002). According to seed type, seed heteromorphism can be classified into di- or polymorphism. In dimorphic seeds, a brown seed is usually larger, non-dormant and can germinate quickly, but the seedling mortality is much higher (a high-risk strategy; Venable 1985*a*) than a black seed. The latter is smaller, dormant and more sensitive to the environment than the brown seed and can enter the seed bank to compensate population size in an unpredictable environment (Venable 1985*a*). Seed heteromorphism is an important strategy for plants to adapt to environmental stress by extending the germination time, minimizing the risk of a single germination and ensuring the successful expansion of the population (Weber 2009).

Besides enhancing the chances of successful germination, seed heteromorphism may also have a significant impact on the growth, physiological characters and stress tolerance of the descendants (Table 1). The seedlings descended from heteromorphic seeds may present significant differences in growth rate and size, which could persist throughout the life of the plant or may disappear in the subsequent stages of development (Wang *et al*. 2010). The non-synchronous germination among heteromorphic seeds usually results in different size of the seedlings. Within a species, although the seeds with relatively larger embryos can grow into larger seedlings than those grown from seeds with smaller embryos, the root-to-shoot ratio is similar between the two, which suggests that the development of heteromorphic plants is in synchrony (Imbert and Ronce 2001). Seed heteromorphism can also have an effect in descendants in response to stress (Table 1).

**Table 1.** Different behaviours between descendants grown from heteromorphic seeds under natural habitat and various stress conditions.

Environment	Species	Seed type	Descendant behaviour	Reference
Natural habitat	*Agropyron psammophilum*	Larger seed/smaller seed	Seedling large/seedling small (difference disappears in 30- to 40-day seedlings but reappear in 50- to 60-day seedlings)	Zhang and Maun (1990)
	*Atriplex triangularis*	Larger seed/smaller seed	Seedling large/seedling small (difference maintained throughout the whole life)	Ellison (1987)
	*Cakile edentula*	Upper seed/lower seed	Disperse long and short distances. Plants from the former were less plastic than those from the latter in response to soil moisture and sand burial	Zhang (1995)
	*Crepis aspera*	Peripheral achene/central achene	Seedling large/seedling small (difference disappears in the later developmental stages)	El-Keblawy (2003)
	*Crepis sancta*	Peripheral achene/central achene	Seedling large/seedling small (not mention how long the difference persisting)	Imbert *et al*. (1996)
	*Desmodium paniculatum*	Larger seed/smaller seed	Seedlings from different seed size have similar relative growth rates	Wulff (1986)
	*Emex spinosa*	Subterranean seed/ aerial seed	Plants from subterranean seeds have larger leaf area and stems than those from aerial achene in mix culture, but these differences do not exist in monoculture when harvested	Weiss (1980)
	*Hedypnois cretica*	Larger seed/smaller seed	Seedling large/seedling small (difference disappears in the later developmental stages)	El-Keblawy (2003)
	*Leontodon saxatilis*	Peripheral achene/central achene	Seedlings from the two morphs did not differ significantly in total biomass	Brändel (2007)
	*Tragopogon pratensis*	Dark seed/pale seed	No obvious difference between both type seedlings	van Mölken *et al*. (2005)
	*Xanthium italicum*	Hypostasis seed/epistasis seed	Seedling large/seedling small (difference appears in the middle stage of development)	Wu *et al*. (2009)
Stress condition	*Chenopodium album*	Black seed/brown seed	Significant difference was observed in plant development and seed proliferation pattern from the two types of seeds only when the parent plants were treated with high salinity	Yao *et al*. (2010)
	*C. sancta*	Peripheral achene/central achene	In intraspecific competition, plants from peripheral achenes dominated those from central achenes; in interspecific competition, the plants from peripheral achenes were advantage at high density; in the absence of competition, both morphs are equally affected by nutrient depletion	Imbert *et al*. (1997)
	*D. paniculatum*	Larger seed/smaller seed	Seedlings from larger seeds have certain advantage when grown together in drought stress, when grown separately, the contrary is the case	Wulff (1986)
	*Heterotheca latifolia*	Disc achene/ray achene	Plants from disc achenes were more successful than ray achenes when a plentiful water supply resulted in a high overall biomass and density; plants from ray achenes were more successful than disc achenes when water was scarce, but overall biomass and density were low	Venable (1985*b*)
	*Suaeda aralocaspica*	Black seed/brown seed	The biomass and mineral profiles of plants from both morph seeds have no obvious difference under different levels of nutrients and salinity	Wang *et al*. (2014)
	*S. salsa*	Black seed/brown seed	Seedling from brown seeds has higher salt tolerance than black seeds; both seedlings have the similar responses to nitrogen availability	Jiang *et al*. (2012)
	*S. splendens*	Black seed/brown seed	Both seedlings have higher salt tolerance, but seedling from brown seed displays less tolerance to lower salinity	Redondo-Gómez *et al*. (2008)
	*T. pratensis*	Dark seed/pale seed	No obvious difference between both type seedlings under nutrient-poor treatment	van Mölken *et al*. (2005)

Previously, the majority of studies have focussed on how environmental factors such as salinity and temperature affect dormancy and germination of heteromorphic seeds (Khan and Ungar 1984; Khan *et al*. 2001*a*, *b*; Wang *et al*. 2008; Aguado *et al*. 2011; Li *et al*. 2011); only a few attempts have been made to investigate the physiological carry-over on descendant plants under stress (Redondo-Gómez *et al*. 2008; Wang *et al*. 2008; Jiang *et al*. 2012). In *Suaeda splendens*, the seedlings derived from both seed morphs showed little difference in growth and photosynthesis in the presence of high salinity, which was the first report on the carry-over of salinity tolerance from different seed morphs to established seedlings and plants (Redondo-Gómez *et al*. 2008). To our knowledge, the first report, which linked physiological responses with differential gene expression in seedlings derived from dimorphic seeds, was from *Atriplex centralasiatica* (Xu *et al*. 2011) and in which a molecular description of differential salt tolerance in dimorphic seeds was provided. In the present study, we extended these findings to responses on salt tolerance of the plants from dimorphic seeds of *S. aralocaspica*, including morphological, physiological and biochemical changes, as well as relevant gene expression profiles.

*Suaeda aralocaspica*, the only species of the *Borszczowia* section of the genus *Suaeda* in the Amaranthaceae, is a monoecious annual halophyte with a single-cell C4 photosynthesis pathway (Voznesenskaya *et al*. 2001) and which is distributed in saline–alkaline sandy soils in the southern margin of Junggar Basin in China (Li 1979; Mao 1994). Plants grow up to 20–50 cm high with unisexual flowers (Commissione Redactorum Florae Xinjiangensis 1994) and can produce heteromorphic seeds with disparate forms and different germination characteristics (Wang *et al*. 2008). The brown seed, with oblate shape and soft seed coat, has a size ∼3.2 × 2.8 × 0.7 mm (length × width × height) and can germinate quickly, while the black seed, with elliptical shape and hard seed coat, has a size ∼2.5 × 2.3 × 1.4 mm and is dormant. To understand the salt tolerance of the descendants grown from the two seed morphs of *S. aralocaspica* and the impact of seed heteromorphism on the progeny, a series of experiments were designed to address the following questions: (i) What are the growth, physiological and biochemical responses of the descendants from dimorphic seeds to long-term NaCl treatment, as well as changes in gene expression pattern? (ii) Is there any different impact of seed heteromorphism on the progeny with or without salinity?

## Methods

### Seed collection

Mature fruits of *S. aralocaspica* were harvested from plants growing in their natural habitat in a desert saline soil at the Wujiaqu 103 regiment (44°29′821″N, 87°31′181″E) in October 2010, in Xinjiang, China. Seeds were air-dried at room temperature, then cleaned and sieved to remove the impurities and stored at 4 °C, 10–12 % relative humidity, in brown paper bags for experiments performed between 2011 and 2012.

### Plant growth and salinity treatment

Seeds of *S. aralocaspica* were sown in pots containing perlite : vermiculite (1 : 3) at a temperature regime of 17–22 °C, 10–20 % relative humidity, a natural light source of 100–500 μmol m^−2^ s^−1^ and a 12–14 h light/10–12 h dark photoperiod. Prior to sowing, the black seeds were stratified according to Wang *et al*. (2008) so that seedling emergence was uniform. In short, the black seeds were placed on two layers of filter paper on top of washed quartz sand, which were moistened with distilled water [water : sand = 1.2 : 10 (w/w)] in metal boxes, which were placed in a refrigerator at 4 °C in the dark for 10 days. Subsequently, the brown and black seeds were sown at the same time. Seedlings were cultivated with half-strength Hoagland (Arnon and Hoagland 1940) solution for 2 months and then treated with half-strength Hoagland solutions containing 100, 300 or 500 mM NaCl for 2 months. To avoid osmotic shock, 300 and 500 mM NaCl treatments were imposed as follows: seedlings were first treated with 100 mM for 12 h, then the NaCl concentration gradually increased to 200, 300 mM or 400, 500 mM every 12 h until the final concentrations were achieved. Thereafter, the final concentration was applied every 2 weeks. For all assays, four samples were collected from young fresh leaves in the upper part of the plant and immediately frozen in liquid nitrogen until use.

### Measurement of growth relevant parameters

The plant height and leaf length were measured with a rectilinear scale, and the stem diameter and leaf width were determined using a Vernier caliper. Ten plants of similar size from the population were used to determine the growth parameters.

### Determination of chlorophyll concentration

Chlorophyll (Chl) concentration was measured in extracts of fresh leaves (0.15 g) with 96 % ethanol as described by Porra *et al*. (1989). The resulting homogenate was centrifuged at 1200*g* for 10 min, and the absorbance of the supernatant recorded (UV-3010, Shimadzu, Japan) at 649 and 665 nm. The Chl_a_ and Chl_b_ concentrations were calculated by the following equations: Chl_a_ = 13.95*D*_665_ − 6.88*D*_649_ and Chl_b_ = 24.96*D*_649_ − 7.32*D*_665_, where *D*_649_ and *D*_665_ are the absorbance values at 649 and 665 nm, respectively. The total Chl concentration in leaves was calculated by the addition of Chl_a_ and Chl_b_.

### Determination of Na^+^ and K^+^ concentrations

Na^+^ and K^+^ measurements were conducted according to Sun *et al*. (2007). Young fresh leaves (1.0 g) from the upper part of the plant were washed three times with distilled water and quickly placed at 105 °C for 10 min to deactivate enzymes, then transferred to an aerated oven at 70 °C for 6–12 h and weighed every 3 h until constant dry weight (DW). Cations were extracted from dry plant material (0.3 g) with HNO_3_ (25 mL), and the Na^+^ and K^+^ concentrations determined by inductively coupled plasma atomic emission spectrometry (Agilent, USA). Pure HNO_3_ was used as control.

### Determination of water content and osmolyte concentration: proline, glycinebetaine, total soluble sugar and total protein

Leaf water content (WC) was measured according to Pujol *et al*. (2001). Young fresh leaves were detached from each treatment and weighed immediately to record the fresh weight (FW). Then the leaves were transferred into an oven at 70 °C drying for 24 h to achieve the DW. The WC was calculated as follows: WC (% FW) = (FW − DW) × 100/FW.

Proline concentration was determined following Zhao *et al*. (2009). Young fresh leaves (0.15 g) were homogenized in aqueous sulfosalicylic acid (5 mL, 3 % (w/v)) and boiled for 10 min (shaking from time to time). A mixture containing leaf extract (200 μL), glacial acetic acid (200 μL) and acidic ninhydrin (300 μL, 2.5 % (w/v)) was incubated in a boiling water bath for 40 min and then cooled on ice. The product was extracted with toluene (500 μL) by vigorous shaking and the absorbance measured at 520 nm; toluene was used as the blank. The concentration of proline was determined by a calibration standard curve (0–20 μg mL^−1^).

Glycinebetaine (GB) was determined according to Huang *et al*. (2009). Young fresh leaves (0.15 g) were ground with distilled water (1.5 mL). After centrifugation at 9000*g* for 15 min, saturated Reinecke's salt (500 μL, 15 mg mL^−1^) was added to the supernatant (300 μL) and the mixture incubated on ice for 1 h, followed by centrifugation at 9000*g* for 15 min. The supernatant was discarded and the precipitate was washed two to three times with 99 % (v/v) ether and then dissolved in acetone (70 %). The absorbance was immediately recorded at 525 nm; a standard curve of GB (0–200 µg mL^−1^) was used to determine the concentration.

The concentration of total soluble sugars (SSs) was determined using the method described by Palma *et al*. (2009). Young fresh leaves (0.12 g) were sheared into pieces and boiled in distilled water (10 mL) for 30 min. Extraction mixture (50 μL) was added to distilled water (150 μL) and reacted with anthrone ethyl acetate (50 μL, 2 % (w/v)). Concentrated sulfuric acid (500 μL) was added to the mixture to develop colour, then immediately boiled for 1 min and cooled in ice water. The absorbance was determined at 630 nm and a standard curve (0–100 µg mL^−1^ of glucose) used to estimate SS.

Total protein concentration was determined by measuring the absorbance at 595 nm according to Bradford (1976). Young fresh leaves (0.1 g) were homogenized in liquid nitrogen and then transferred into a 1.5-mL micro-tube with extraction buffer (500 μL, 12.5 mM Tris–Cl, pH 6.8). The homogenate was centrifuged at 10 000*g* for 15 min at 4 °C. All of the antioxidant enzyme activities were expressed as unit per mg total protein.

### Measurement of ${O_{2}}^{-}$ production, H_2_O_2_ concentration and lipid peroxidation

${{\text{O}}_{2}}^{-}$ production was estimated according to the method described by Li and Gong (2005). Young fresh leaves (0.2 g) were homogenized with sodium phosphate buffer (phosphate-buffered saline (PBS), 50 mM, pH 7.8) on ice and the mixture centrifuged at 10 000*g* for 20 min at 4 °C. The supernatant (1 mL) was then incubated with hydroxylamine (1 mL, 1 mM) at 25 °C for 1 h, then 4-aminobenzenesulfonic acid (1 mL, 17 mM) and α-naphthalenamine (1 mL, 7 mM) were added and the test tubes incubated at 25 °C for 20 min. Reaction mixture (500 µL) was combined with an equal volume of ether to eliminate the pigments. The mixture was centrifuged at 9000*g* for 3 min and the absorbance of the supernatant recorded at 530 nm. A standard curve using NaNO_2_ was plotted within the range of 0–50 μM.

The H_2_O_2_ concentration was determined by a method described by Sun *et al*. (2010) with some minor changes. Young fresh leaves (0.15 g) were homogenized with cooled acetone and the homogenate was centrifuged at 1000*g* for 10 min at 4 °C. Then supernatant (1 mL) was transferred into a new 1.5-mL micro-tube followed by the addition of titanium sulfate (100 μL, 5 % (w/v)) and ammonia (200 μL) to precipitate the titanium–hydroperoxide complex, followed by centrifugation as above. The supernatant was discarded and the precipitate suspended and washed three to five times with acetone to remove the pigments and then dissolved in sulfuric acid (5 mL, 2 M). The absorbance was measured at 415 nm and compared with a standard hydrogen peroxide curve (0–100 µM of H_2_O_2_).

Lipid peroxidation was determined by measuring the concentration of malondialdehyde (MDA) produced by the thiobarbituric acid (TBA) reaction as described by Heath and Packer (1968). Young fresh leaves (0.12 g) were ground in trichloroacetic acid (TCA, 500 µL, 612 mM). Trichloroacetic acid (700 µL, 612 mM) was added to the homogenate, which was centrifuged at 1200*g* for 10 min. The supernatant (300 µL) was mixed with an equal volume of TBA (34.7 mM), boiled for 10 min, cooled in an ice-bath and then centrifuged at 12 000*g* for 10 min. The absorbance of the supernatant was monitored at 532, 450 and 600 nm. The concentration of MDA (*C*) was calculated by using the following equation: *C* = 6.45 × (*D*_532_ − *D*_600_) − 0.56 × *D*_450_, where *D*_450_, *D*_532_ and *D*_600_ represent absorbance values at 450, 532 and 600 nm, respectively, and the results expressed as µmol g^−1^ DW.

### Determination of non-enzymatic antioxidant concentrations: ascorbic acid and glutathione

The ascorbic acid (AsA) concentration was determined by a method described by Hamed *et al*. (2007), which is based on the reduction of Fe^3+^ to Fe^2+^ by AsA in acid solution and the spectrometric detection of Fe^2+^ complexed with 2,2-dipyridyl at 525 nm. Young fresh leaves (0.15 g) were homogenized with ice-cold TCA (500 µL, 306 mM) and the mixture centrifuged at 12 000*g* for 10 min at 4 °C. The extract (supernatant) was transferred into a reaction solution, containing NaH_2_PO_4_ (100 µL, 150 mM), TCA (200 µL, 612 mM), H_3_PO_4_ (200 µL, 4.49 M), FeCl_3_ (100 µL, 184.9 mM) and 2,2-biphenyl (200 µL, 214.8 mM), then incubated at 37 °C for 60 min. A standard curve covering the range of 0–70 mM AsA was plotted.

For the glutathione (GSH) assay, tissue extract (as that in the AsA assay) was mixed with NaH_2_PO_4_ (130 µL, 150 mM) and 5,5-dithio-bis(2-nitrobenzoic acid) (90 µL). The mixture was incubated at 30 °C for 5 min, and the absorbance at 412 nm was recorded (Otto and Moon 1996). The total GSH was calculated from a standard curve prepared with 0–0.12 mM GSH solution.

### Measurement of the activities of antioxidant enzymes: superoxide dismutase, peroxidase, catalase, ascorbate peroxidase and glutathione reductase

Young fresh leaves (0.15 g) were homogenized in ice-cold buffer (1.5 mL) containing PBS (pH 7.8, 50 mM), EDTA (0.5 mM), polyvinylpyrrolidone (1 %), AsA (1 mM) and glycerol (10 %). The homogenate was filtered and centrifuged at 10 000*g* for 15 min at 4 °C. The supernatant was transferred onto ice as crude enzyme and immediately used for analysis of the activity.

Superoxide dismutase (SOD) activity was determined by measuring its ability to inhibit the photochemical reduction of nitroblue tetrazolium (NBT) chloride, as described by Basu *et al*. (2010). Crude enzyme extract (50 µL) was added into test tubes with reaction mixture (3 mL) consisting of PBS (pH 7.8, 50 mM), EDTA-Na_2_ (100 µM), methionine (130 mM), NBT (750 µM) and riboflavin (2 µM); the test tubes were incubated at 25 °C under a light source of 72 μmol m^−2^ s^−1^ for 25 min. As blank treatment, an aluminium foil wrapped blank tube was employed. The absorbance of the reaction mixture was read at 560 nm. One unit (U) of SOD activity was defined as the amount of enzyme required to cause 50 % inhibition of the NBT photoreduction rate.

Peroxidase (POD) and catalase (CAT) activities were defined as guaiacol increase at 470 nm or H_2_O_2_ decrease at 240 nm, respectively, according to the method of Abogadallah *et al*. (2010). For POD analysis, crude enzyme solution (500 µL) was added into reaction mixture (3 mL) containing H_2_O_2_ (1 mL, 0.3 %), guaiacol (950 µL, 0.2 %) and PBS (pH 7.0, 1 mL). For CAT measurement, crude enzyme extract (100 µL) was added into reaction mixture (3 mL) containing H_2_O_2_ (1 mL, 0.3 %) and H_2_O (1.9 mL) to initiate the reaction. The absorbance values of the POD and CAT mixtures were recorded within 1 min (UV-3010, Shimadzu), against a blank without the crude enzyme.

Ascorbate peroxidase (APX) activity was assayed according to Nakano and Asada (1981) with a minor modification. The reaction mixture (3 mL) contained PBS (pH 7.0, 50 mM), ascorbate (15 mM), H_2_O_2_ (0.3 mM) and enzyme extract (100 µL), and the activity was indicated by the change in absorbance at 290 nm. The values were calculated in terms of micromoles of ascorbate oxidized per minute.

Glutathione reductase (GR) activity was determined by the method of Foyer and Halliwell (1976), with minor modifications, based on the oxidation of NADPH at 340 nm for 1 min in a reaction mixture (1 mL) containing NADPH (1 mM), Tricine–NaOH (pH 7.8, 100 mM), glutathione disulfide (5 mM) and enzyme extract (200 µL). Corrections were made by subtraction of the background absorbance at 340 nm in the absence of NADPH.

### Estimation of the activities of key photosynthetic enzymes: phosphoenolpyruvate carboxylase and ribulose-1,5-bisphosphate carboxylase/oxygenase

For preparation of the crude enzyme of phosphoenolpyruvate carboxylase (PEPC), young fresh leaves (0.4 g) were homogenized on ice in extraction buffer (1.5 mL) containing Tris–H_2_SO_4_ (pH 8.2, 100 mM), β-mercaptoethanol (7 mM), EDTA (1 mM) and glycerol (5 %) (Zhang and Qu 2005). The homogenate was centrifuged at 2000*g* for 20 min at 4 °C and the supernatant used immediately for assay of the activity of PEPC. The reaction mixture (1 mL) consisted of MgSO_4_ (143 µL, 70 mM), NaHCO_3_ (143 µL, 70 mM), phosphoenolpyruvic acid (286 µL, 14 mM) and NADH (429 µL, 5 mM), followed by incubation at 30 °C for 30 min; finally, malate dehydrogenase (66 U) and crude enzyme solution (40 µL) were added into the mixture to initiate the reaction.

For the activity of ribulose-1,5-bisphosphate carboxylase/oxygenase (RUBPC) assay, young fresh leaves (0.5 g) were ground on ice in extraction buffer (2.5 mL) containing Tris–HCl (pH 7.6, 40 mM), MgCl_2_ (10 mM), EDTA (0.25 mM) and GSH (5 mM) (Zhang and Qu 2005). The homogenate was then centrifuged at 2000*g* for 15 min at 4 °C and the supernatant used immediately for assay of RUBPC activity. The reaction mixture (1 mL) consisting of NaHCO_3_ (67 µL, 0.2 mM), reaction buffer (467 µL) [Tris–HCl (pH 7.8, 100 mM), MgCl_2_ (12 mM), EDTA (0.4 mM)] and crude enzyme extract (133 µL) was incubated at 30 °C for 10 min, then the following were added: NADH (67 µL, 5 mM), ATP (67 µL, 50 mM), phosphocreatine (67 µL, 50 mM), creatine phosphate kinase (33 µL, 160 U mL^−1^), phosphoglycerate kinase (33 µL, 160 U mL^−1^), phosphoglyceraldehyde dehydrogenase (33 µL, 160 U mL^−1^) and RuBP (33 µL, 25 mM) to initiate the reaction. The blank was set in the absence of RuBP.

The absorbance was recorded for 3 min at 340 nm. Enzyme activities of PEPC and RUBPC were defined as 0.01 optical density value decrease per minute is 1U.

### Quantitative real-time polymerase chain reaction analysis of gene expression

Total RNA was extracted from young fresh leaves (0.1 g) using the RNAprep Pure Plant Kit (Tiangen, Beijing, China). Each reverse transcription reaction was performed with total RNA (1.5 μg) using the Quant First Strand cDNA Synthesis Kit (Tiangen) according to the manufacturer's instruction. Based on the published sequences, conserved sequences of 10 target genes and the internal control of *β-actin* were employed to design degenerate primers of each gene. The subsequent polymerase chain reaction (PCR) products were sequenced with the gene-specific primers for quantitative real-time (qRT)-PCR that were generated (Table 2).

**Table 2.** Gene-specific primers of target genes used in RT-PCR analysis. *BADH*, betaine aldehydrogenase gene; *CAT*, catalase gene; *cSOD*, cytoplasm Cu/Zn SOD gene; *GR*, glutathione reductase gene; *P5CS*, pyrroline-5-carboxylate synthase gene; *PEPC*, phosphoenolpyruvate carboxylase gene; *RUBPC*, ribulose-1,5-bisphosphate carboxylase/oxygenase gene; *sAPX*, chloroplast stromal APX gene; *V-ATPase*, vacuolar ATP synthase gene; *V-PPase*, vacuolar pyrophosphatase gene; *β-actin*, internal reference gene.

Primer names	Primer sequences (5′→3′)	
	Forward	Reverse
*BADH*	GGAGTTGGCATCTGTGACTTGTCTAG	CCATTCAGCAGCTACGTCAAGGTC
*CAT*	GTTCGATTCTCCACTGTTATTCATGAAC	AGTGAACATGTGCAAACTCTCAGGAT
*cSOD*	GTGACACAACTAATGGATGCATGTC	AGTTCATGTCCACCTCTTCCAAGAT
*GR*	GACAAGAGTGGAGCCATAGAGGTG	CAGATGGAATAGCTCGATAATCAGGT
*P5CS*	CGATGCAGTAAGTACCAGGAAAGCTC	ATGAGACCATTCTGCCCAACAGC
*PEPC*	GACCCAGGAATCGCTGCTTTATATGAC	CACTGTTACATGGAAGTCAGGATCACG
*RUBPC*	ACGGTCGAGCAGTTTATGAATGTC	GTCTTCACATGTACCCGCAGTAGC
*sAPX*	CATATGCAGATCTATTTCAGTTGGCTA	CAATGTATGTGCTCCAGATAATGC
*V-ATPase*	ATCTGGCCAATGACCCTACAATTGAG	CGTTCATAGATGGTAGCCAAATCTGTG
*V-PPase*	AGCTGTTATCGCTGACAATGTTGGTG	GTCGGTTGCAAACAAGGTAGTGATC
*β-actin*	CCAAAGGCCAACAGAGAGAAGAT	TGAGACACACCATCACCAGAAT

For PCR ampliﬁcation, the reactions were ﬁrst incubated for 2 min at 95 °C, followed by 40 cycles of 30 s at 95 °C, 30 s at 56–62 °C (annealing temperature was calculated according to the melting temperature of each pair of gene-speciﬁc primers), 50 s at 72 °C, then for 10 min extension at 72 °C. Three samples (biological replicates) per gene were duplicated (technical replicates) in qRT-PCR experiment.

### Statistical analysis

All data were analysed using the software of GraphPad Prism Version 4.02 for Windows (GraphPad Software, San Diego, CA, USA). Data were subjected to unpaired *t*-test for independent samples. Two-way analyses of variance were used to test the significance of main effects between seed type and salinity and their interactions. If significant main effects existed, differences were tested by a multiple comparison Tukey test at the 0.05, 0.01 and 0.001 significance level.

## Results

### Changes of plant growth in descendants of heteromorphic seeds under saline conditions

**Figure 1.**
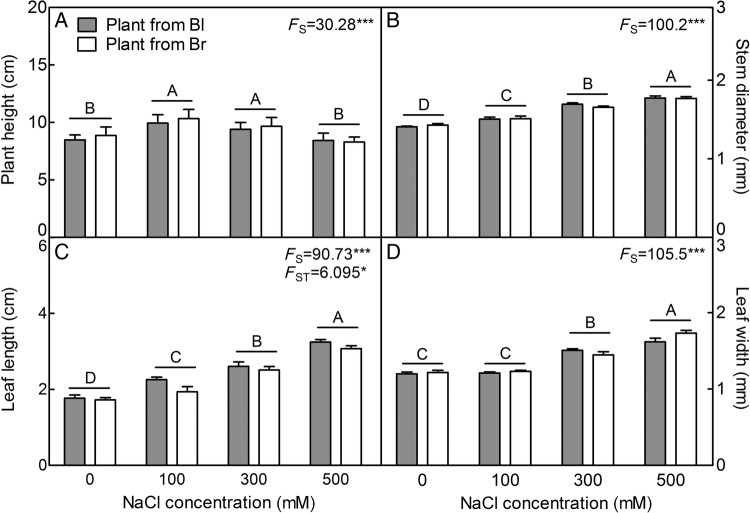
Responses of plant growth to long-term NaCl treatment. (A) Plant height, (B) stem diameter, (C) leaf length and (D) leaf width. Two-month-old plants were treated with various NaCl concentrations for 2 months before measurement. The growth conditions were 17–22 °C, 10–20 % relative humidity, 100–500 μmol m^−2^ s^−1^ light source and a 12–14 h light/10–12 h dark photoperiod. *F*-values are given when significance levels are reached (S, salt; ST, seed type; *P<0.05, ***P< 0.001). Bars with different uppercase letters indicate signiﬁcant differences (*P*< 0.05) according to Tukey's test. Values are means ± SE of 10 plants. Bl, black seed; Br, brown seed.

Plants grown from dimorphic seeds in 100 and 300 mM NaCl treatments were significantly taller than those grown in the absence of NaCl (*F*_3,72_ = 30.28, *P*< 0.0001) (Fig. 1A). In addition, with the increase of NaCl concentration, the stem diameter (*F*_3,72_ = 100.2, *P*< 0.0001) (Fig. 1B), leaf length (*F*_3,72_ = 90.73, *P*< 0.0001) (Fig. 1C) and width (*F*_3,72_ = 105.5, *P*< 0.0001) (Fig. 1D) were significantly increased in plants from both types of seeds compared with those in the absence of salt. For all of the growth indexes, plants derived from two seed types showed no significant difference (*F*_1,72_ = 2.334, *P* = 0.1310 for plant height; *F*_1,72_ = 0.09851, *P* = 0.7545 for stem diameter and *F*_1,72_ = 1.135, *P* = 0.2902 for leaf width, respectively) under various NaCl concentrations, except for leaf length (*F*_1,72_ = 6.095, *P* = 0.0159).

### Effect of long-term salt treatment on physiological responses and relevant gene expressions in plants from heteromorphic seeds

#### Changes of inorganic and organic osmolytes

**Figure 2.**
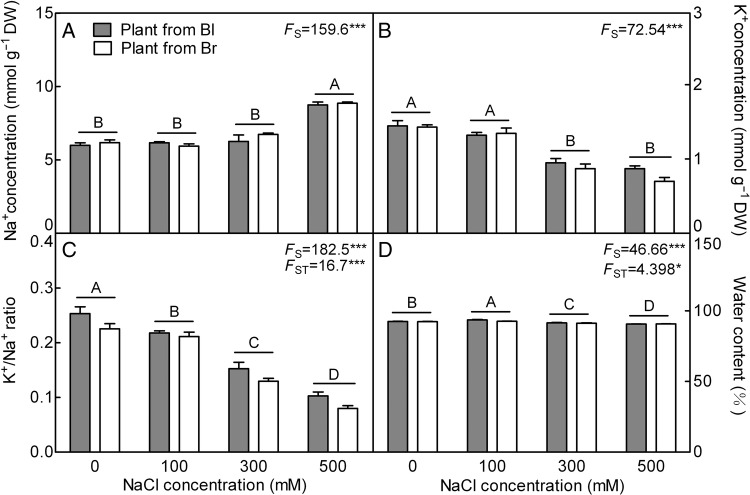
Changes of Na^+^, K^+^ and WC in leaves during long-term NaCl treatment. (A) Na^+^, (B) K^+^, (C) K^+^/Na^+^ ratio and (D) WC. *F*-values are given when significance levels are reached (S, salt; ST, seed type; *P< 0.05, ***P< 0.001). Bars with different uppercase letters indicate signiﬁcant differences (*P*< 0.05) according to Tukey's test. Values are means ± SE of four replicates. Bl, black seed; Br, brown seed.

With increasing salt concentration, plants from the two seed morphs showed a significantly greater accumulation of Na^+^ in the leaf than that of plants grown without salt (*F*_3,24_ = 159.6, *P*< 0.0001) (Fig. 2A). On the other hand, salt treatment induced a significant decrease of K^+^, which was greater at 300 and 500 mM NaCl treatments than at lower concentrations (*F*_3,24_ = 72.54, *P*< 0.0001) (Fig. 2B). As a consequence, the K^+^/Na^+^ ratios in both plants were reduced (*F*_3,24_ = 182.5, *P*< 0.0001) (Fig. 2C). Similarly, the WC was affected with elevated salt level (*F*_3,24_ = 46.66, *P*< 0.0001) (Fig. 2D). Plants from both types of seeds showed no significant difference (*F*_1,24_ = 1.728, *P* = 0.2011 for Na^+^; *F*_1,24_ = 2.524, *P* = 0.1252 for K^+^) in Na^+^ and K^+^ concentrations, although both the K^+^/Na^+^ ratios (*F*_1,24_ = 16.7, *P* = 0.0004) and WC (*F*_1,24_ = 4.398, *P* = 0.0467) did differ between seed morphs.

**Figure 3.**
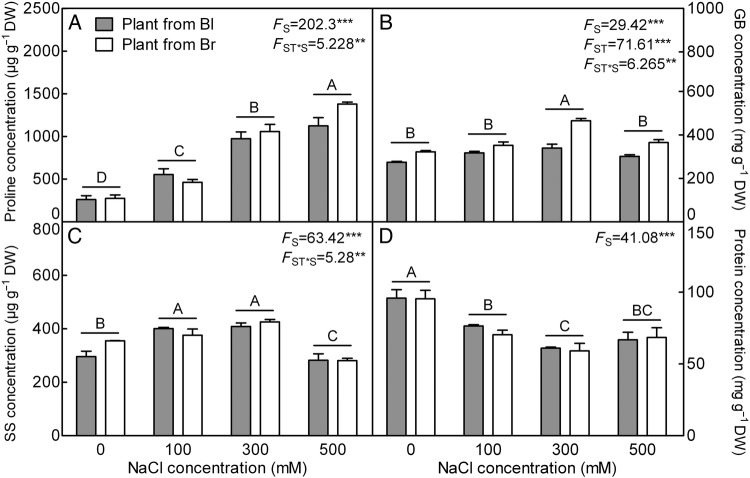
Changes of osmolyte concentration in leaves during long-term NaCl treatment. (A) Proline, (B) GB, (C) SS and (D) total soluble protein. *F*-values are given when significance levels are reached (S, salt; ST, seed type; **P< 0.01, ***P< 0.001). Bars with different uppercase letters indicate signiﬁcant differences (*P*< 0.05) according to Tukey's test. Values are means ± SE of four replicates. Bl, black seed; Br, brown seed.

A significant increase of proline concentration was observed in descendants of the two types of seeds, when grown in 300 and 500 mM NaCl (∼2- to 3-fold higher compared with plants grown in the absence of salt) (*F*_3,24_ = 202.3, *P*< 0.0001) (Fig. 3A). With the increasing NaCl concentration, the GB (*F*_3,24_ = 29.42, *P*< 0.0001) and SS (*F*_3,24_ = 63.42, *P*< 0.0001) concentrations increased significantly at 300 mM NaCl concentration (Fig. 3B and C), while the total soluble protein (*F*_3,24_ = 41.08, *P*< 0.0001) decreased significantly (Fig. 3D). In comparison between different plant types, the response of proline (*F*_1,24_ = 4.255, *P* = 0.0501), SS (*F*_1,24_ = 2.628, *P* = 0.1180) and protein (*F*_1,24_ = 0.5051, *P* = 0.4841) concentrations did not differ under the various NaCl concentrations, while the accumulations of GB (*F*_1,24_ = 71.61, *P*< 0.0001) in plants from brown seeds were significantly greater at higher NaCl concentration than that of the plants from black seed. Only GB concentration was significantly affected by the interaction of seed type and salinity concentration (*F*_3,24_ = 6.265, *P* = 0.0027).

#### Expression pattern of ion-transport- and osmotic-regulation-related genes

**Figure 4.**
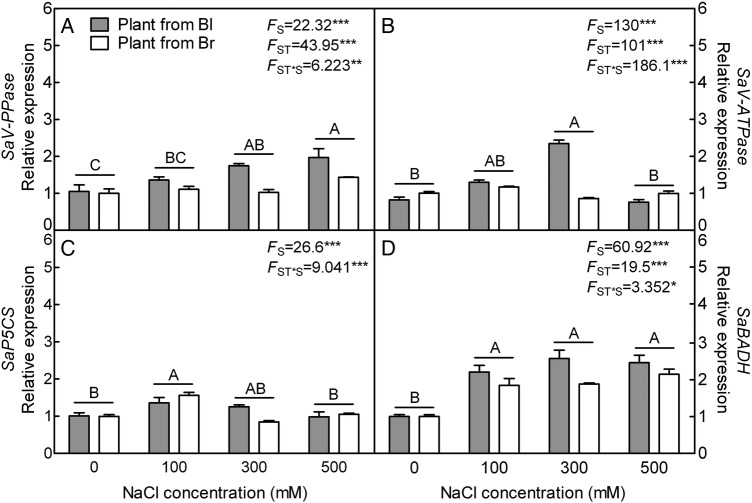
qRT-PCR analysis of gene expression pattern on ion transportation and osmolyte synthesis in leaves during long-term NaCl treatment. (A) *V-PPase*, (B) *V-ATPase*, (C) pyrroline-5-carboxylate synthase gene and (D) *BADH* gene. *β-actin* as internal reference. *F*-values are given when significance levels are reached (S, salt; ST, seed type; *P< 0.05, **P< 0.01, ***P< 0.001). Bars with different uppercase letters indicate signiﬁcant differences (*P*< 0.05) according to Tukey's test. Values are means ± SE of six replicates (including three biological replicates and two technical replicates). Bl, black seed; Br, brown seed.

The expression level of vacuolar pyrophosphatase gene (*V-PPase*) was significantly increased under 300 and 500 mM NaCl treatment in plants from brown and black seeds (*F*_3,40_ = 22.32, *P*< 0.0001) (Fig. 4A), while that of the key subunit B of vacuolar ATP synthase gene (*V-ATPase*) remained unchanged except for the plants from black seeds under 300 mM NaCl treatment (*F*_3,40_ = 130.0, *P*< 0.0001) (Fig. 4B). In comparison between the plants derived from the two types of seeds, an apparent difference was observed in the transcript accumulation of *V-PPase* (*F*_1,40_ = 43.95, *P*< 0.0001) and *V-ATPase* (*F*_1,40_ = 101.0, *P*< 0.0001) at 300 mM NaCl treatment: expression was significantly higher in plants from black seed compared with that of plants from brown seed (*t*_10_ = 11.40, *P*< 0.0001 for *V-PPase*; *t*_10_ = 29.65, *P*< 0.0001 for *V-ATPase*).

The expression level of pyrroline-5-carboxylate synthase gene (*P5CS*) was significantly increased under 100 mM NaCl treatment in plants from heteromorphic seeds (*F*_3,40_ = 26.60, *P*< 0.0001) (Fig. 4C). There was no significant difference between two types of plants (*F*_1,40_ = 0.6608, *P* = 0.4211). The transcript accumulation of betaine aldehyde dehydrogenase (*BADH*) gene was significantly increased under various NaCl concentrations in plants from both types of seeds compared with plants grown in the absence of salt (*F*_3,40_ = 60.92, *P*< 0.0001), which was obviously affected by seed type (*F*_1,40_ = 19.50, *P*< 0.0001) and significantly higher in plants from black seed at 300 mM NaCl than that of brown seed (*t*_10_ = 4.118, *P* = 0.0021) (Fig. 4D).

### Changes of antioxidant enzyme and non-enzymatic antioxidant and expression pattern of relevant genes

#### Oxidative stress level

**Figure 5.**
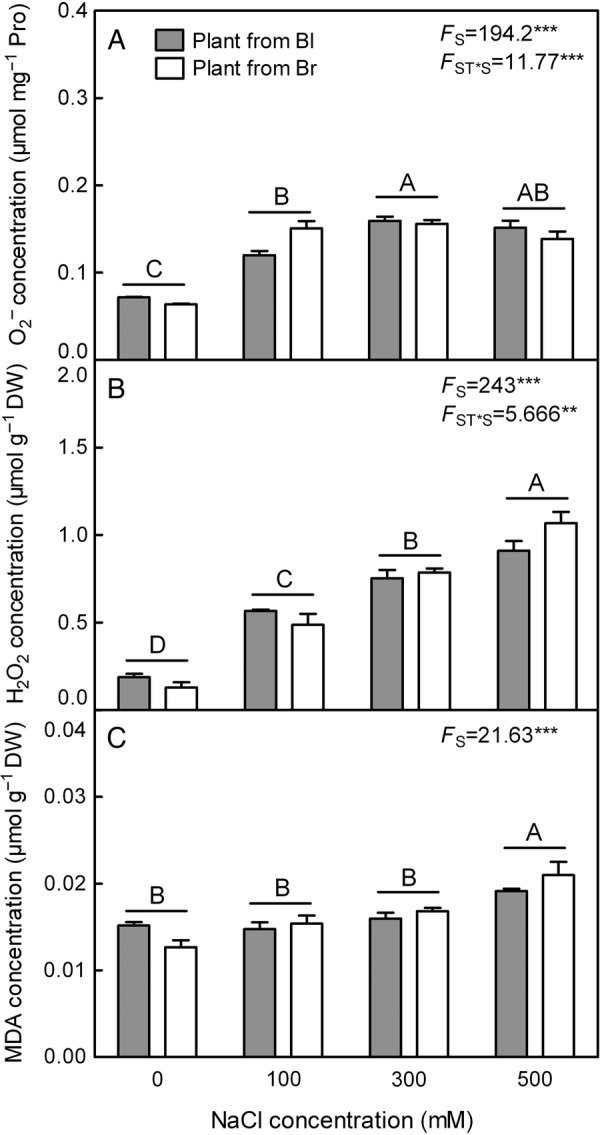
Changes of ROS level and MDA concentration in leaves during long-term NaCl treatment. (A) ${{\text{O}}_{2}}^{-}$ production rate, (B) hydrogen peroxide concentration, (C) MDA concentration. *F*-values are given when significance levels are reached (S, salt; ST, seed type; **P< 0.01, ***P< 0.001). Bars with different uppercase letters indicate signiﬁcant differences (*P*< 0.05) according to Tukey's test. Values are means ± SE of four replicates. Bl, black seed; Br, brown seed.

${{\text{O}}_{2}}^{-}$ level (*F*_3,24_ = 194.2, *P*< 0.0001) and H_2_O_2_ production (*F*_3,24_ = 243.0, *P*< 0.0001) in both types of plants from heteromorphic seeds were significantly enhanced by salt treatment (Fig. 5A and B). Corresponding to the rising ${{\text{O}}_{2}}^{-}$ and H_2_O_2_ levels, MDA concentration (*F*_3,24_ = 21.63, *P*< 0.0001) was significantly higher than that of plants grown without salt at 500 mM NaCl treatment in both types of plants (Fig. 5C). No significant difference (*F*_1,24_ = 0.3256, *P* = 0.5735 for ${{\text{O}}_{2}}^{-};$ *F*_1,24_ = 0.3422, *P* = 0.5640 for H_2_O_2_; *F*_1,24_ = 0.1253, *P* = 0.7265 for MDA) was observed between two types of plants in the above indexes, while ${{\text{O}}_{2}}^{-}$ level (*F*_3,24_ = 11.77, *P*< 0.0001) and H_2_O_2_ concentration (*F*_3,24_ = 5.666, *P* = 0.0044) were significantly affected by the interaction of seed type and salt concentration.

#### Antioxidant enzymes and non-enzymatic antioxidants

**Figure 6.**
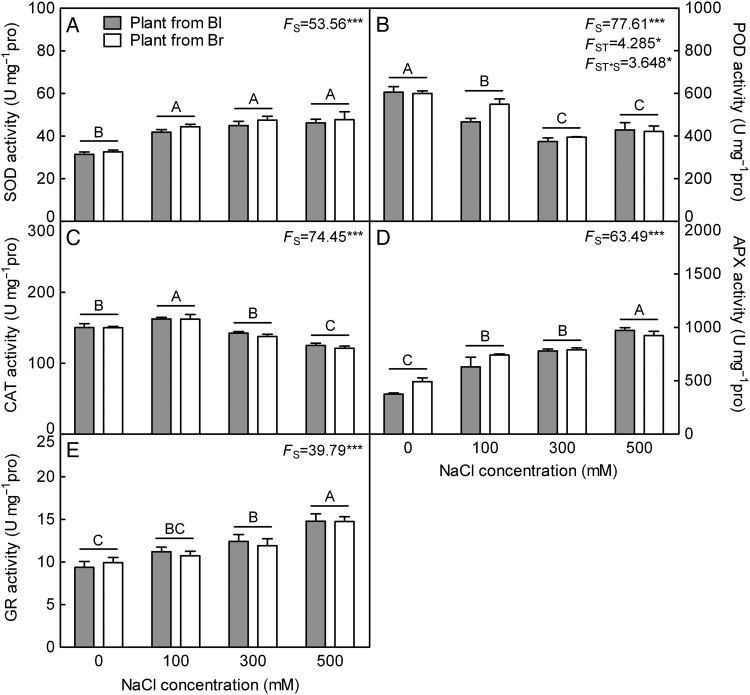
Changes of the activity of antioxidant enzyme in leaves during long-term NaCl treatment. (A) SOD, (B) POD, (C) CAT, (D) APX and (E) GR. *F*-values are given when significance levels are reached (S, salt; *P< 0.05, ***P< 0.001). Bars with different uppercase letters indicate signiﬁcant differences (*P*< 0.05) according to Tukey's test. Values are means ± SE of four replicates. Bl, black seed; Br, brown seed.

The activities of SOD (*F*_3,24_ = 53.56, *P*< 0.0001), APX (*F*_3,24_ = 63.49, *P*< 0.0001) and GR (*F*_3,24_ = 39.79, *P*< 0.0001) increased significantly in response to NaCl treatment, especially at higher salt concentrations (300 and 500 mM NaCl) (Fig. 6A, D and E), while significant decreases were measured for POD (*F*_3,24_ = 77.61, *P*< 0.0001) and CAT (*F*_3,24_ = 74.45, *P*< 0.0001) activities, particularly under 300 or 500 mM NaCl treatment (Fig. 6B and C). For all indexes of enzyme activity, no significant difference was detected between plants derived from two seed types under different NaCl concentrations (*F*_1,24_ = 4.143, *P* = 0.053 for SOD; *F*_1,24_ = 3.119, *P* = 0.0901 for APX; *F*_1,24_ = 0.1041, *P* = 0.7497 for GR; *F*_1,24_ = 1.408, *P* = 0.2471 for CAT), except for POD (*F*_1,24_ = 4.285, *P* = 0.0494).

**Figure 7.**
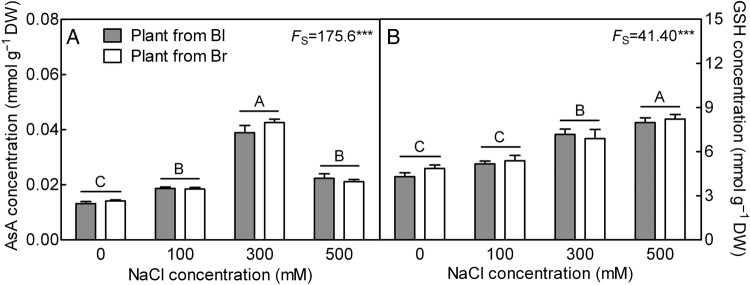
Changes of non-enzymatic antioxidant concentration in leaves during long-term NaCl treatment. (A) Ascorbic acid and (B) GSH. *F*-values are given when significance levels are reached (S, salt; ***P< 0.001). Bars with different uppercase letters indicate signiﬁcant differences (*P*< 0.05) according to Tukey's test. Values are means ± SE of three replicates. Bl, black seed; Br, brown seed.

With the increasing NaCl concentration, the AsA (*F*_3,16_ = 175.6, *P*< 0.0001) and GSH (*F*_3,16_ = 41.40, *P*< 0.0001) increased significantly in both plant types and reached to their highest values at 300 and/or 500 mM NaCl concentrations (Fig. 7A and B), although a significant decrease in AsA concentration was observed at 500 mM compared with 300 mM NaCl treatment (*t*_10_ = 10.89, *P*< 0.0001), it was still significantly higher than that of plants grown in the absence of salt (Fig. 7A). There was no significant difference in antioxidant concentration between plants from brown and black seeds (*F*_1,16_ = 0.8069, *P* = 0.3824 for AsA; *F*_1,16_ = 0.5116, *P* = 0.4848 for GSH).

#### Gene expression pattern of antioxidant enzymes and non-enzymatic antioxidants

**Figure 8.**
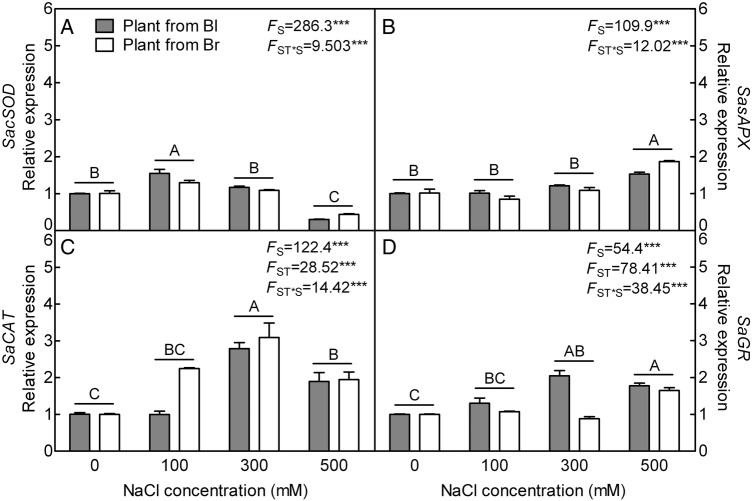
qRT-PCR analysis of gene expression pattern on antioxidant enzyme in leaves during long-term NaCl treatment. (A) Cytoplasm Cu/Zn superoxide dismutase gene, (B) chloroplast stromal ascorbate peroxidase gene, (C) *CAT* gene and (D) *GR* gene. *β-actin* as internal reference. *F*-values are given when significance levels are reached (S, salt; ST, seed type; ***P< 0.001). Bars with different uppercase letters indicate signiﬁcant differences (*P*< 0.05) according to Tukey's test. Values are means ± SE of six replicates (including three biological replicates and two technical replicates). Bl, black seed; Br, brown seed.

The expression level of *sAPX* (chloroplast stromal APX) (*F*_3,40_ = 109.9, *P*< 0.0001), *CAT* (*F*_3,40_ = 122.4, *P*< 0.0001) and *GR* (*F*_3,40_ = 54.40, *P*< 0.0001) increased significantly under 300 or 500 mM NaCl treatment in both types of plants (Fig. 8B–D), while *cSOD* (cytoplasmic Cu/Zn SOD) was up-regulated at 100 mM, but a significant reduction was observed at 500 mM NaCl treatment compared with that without salt (*F*_3,40_ = 286.3, *P*< 0.0001) (Fig. 8A). Some differences were observed between plants from two types of seeds. In plants from black seed, the transcript accumulations of *cSOD* at 100 mM (*t*_10_ = 2.737, *P* = 0.0209) and *GR* at 300 mM (*t*_10_ = 10.64, *P*< 0.0001) were significantly higher than that of plants from brown seed; in plant from brown seed, the expression level of *CAT* at 100 mM (*t*_10_ = 18.45, *P*< 0.0001) and *sAPX* at 500 mM (*t*_10_ = 9.145, *P*< 0.0001) was significantly higher than that of plants from black seed. All of the above indexes were significantly affected by the interaction of seed type and salt concentration (*F*_3,40_ = 9.503, *P*< 0.0001 for *cSOD*; *F*_3,40_ = 12.02, *P*< 0.0001 for *sAPX*; *F*_3,40_ = 14.42, *P*< 0.0001 for *CAT*; *F*_3,40_ = 38.45, *P*< 0.0001 for *GR*).

### Changes of Chl and activity of key photosynthesis enzymes

**Figure 9.**
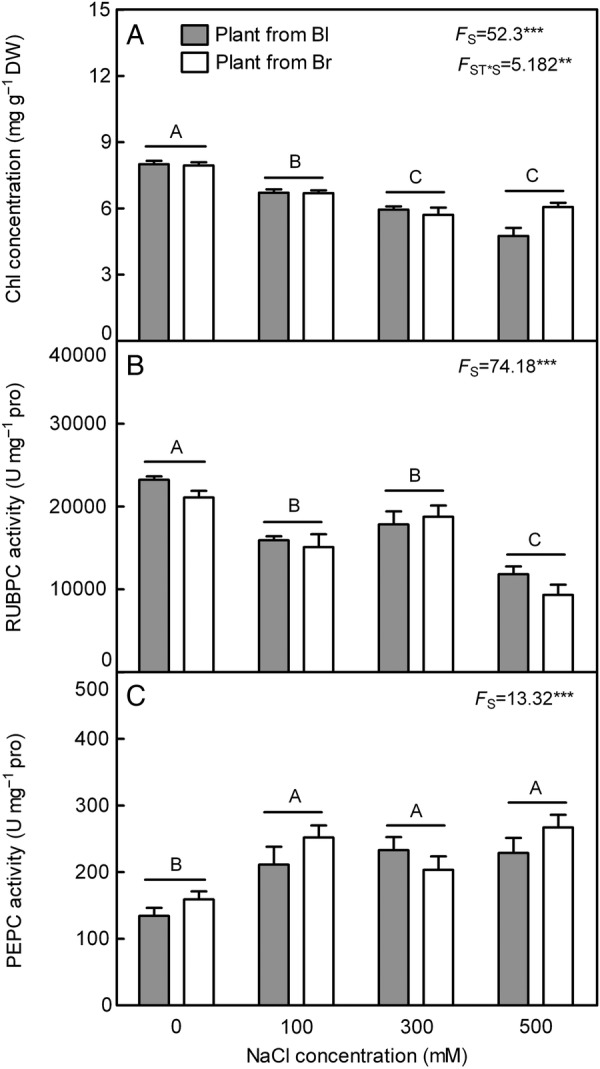
Changes of Chl concentration and activity of photosynthesis enzymes in leaves during long-term NaCl treatment. (A) Chl, (B) ribulose-1,5-bisphosphate carboxylase and (C) PEPC. *F*-values are given when significance levels are reached (S: salt; ST: seed type; **P< 0.01, ***P< 0.001). Bars with different uppercase letters indicate signiﬁcant differences (*P*< 0.05) according to Tukey's test. Values are means ± SE of four replicates. Bl, black seed; Br, brown seed.

The Chl concentration decreased significantly with the increase in salt concentration in both types of plants (*F*_3,24_ = 52.30, *P*< 0.0001) (Fig 9A). The activities of RUBPC and PEPC displayed a contrary pattern in response to salt treatment (Fig. 9B and C). Activity of ribulose-1,5-bisphosphate carboxylase/oxygenase decreased significantly with the increasing salt concentration (*F*_3,24_ = 74.18, *P*< 0.0001) (Fig. 9B), while salinity significantly stimulated activity of PEPC (*F*_3,24_ = 13.32, *P*< 0.0001) (Fig. 9C). No obvious difference was observed between plants from two types of seeds in total Chl concentration (*F*_1,24_ = 2.502, *P* = 0.1268) and the activities of two key photosynthesis enzymes (*F*_1,24_ = 3.975, *P* = 0.0577 for RUBPC; *F*_1,24_ = 2.311, *P* = 0.1415 for PEPC), while Chl concentration (*F*_3,24_ = 5.182, *P* = 0.0067) was significantly affected by the interaction of seed type and salt concentration.

### Gene expression pattern of PEPC and RUBPC

**Figure 10.**
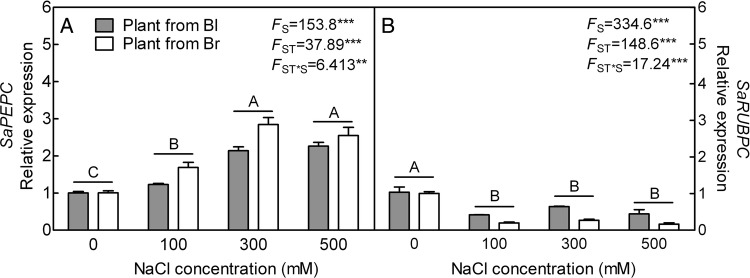
qRT-PCR analysis of gene expression pattern on *PEPC* and *RUBPC* in leaves during long-term NaCl treatment. (A) *PEPC* gene and (B) Ribulose-1,5-bisphosphate carboxylase gene. *β-actin* as internal reference. *F*-values are given when significance levels are reached (S, salt; ST, seed type; **P< 0.01, ***P< 0.001). Bars with different uppercase letters indicate signiﬁcant differences (*P*< 0.05) according to Tukey's test. Values are means ± SE of six replicates (including three biological replicates and two technical replicates). Bl, black seed; Br, brown seed.

With the increase in salt concentration, *PEPC* was significantly up-regulated (*F*_3,40_ = 153.8, *P*< 0.0001) (Fig. 10A), while *RUBPC* was significantly decreased (*F*_3,40_ = 334.6, *P*< 0.0001) (Fig. 10B) in both types of plants compared with plants grown without salt. Significant differences between plants from the two types of seeds were observed (*F*_1,40_ = 37.98, *P*< 0.0001 for *PEPC*; *F*_1,40_ = 148.6, *P*< 0.0001 for *RUBPC*). In plants from brown seed, the transcript accumulation of *PEPC* at 100 mM (*t*_10_ = 4.448, *P* = 0.0012) and 300 mM (*t*_10_ = 4.304, *P* = 0.0016) was significantly higher than that of plants from black seed; in plants from black seed, the *RUBPC* expression level was significantly higher than that of plant from brown seed under various salt concentrations (*t*_10_ = 11.27, *P*< 0.0001 for 100 mM; *t*_10_ = 17.25, *P*< 0.0001 for 300 mM; *t*_10_ = 6.685, *P*< 0.0001 for 500 mM). Both of above indexes was significantly affected by the interaction of seed type and salt concentration (*F*_3,40_ = 6.413, *P* = 0.0012 for *PEPC*; *F*_3,40_ = 17.24, *P*< 0.0001 for *RUBPC*). The expression patterns of *PEPC* and *RUBPC* agreed with the enzyme activities of PEPC and RUBPC, respectively.

## Discussion

*Suaeda aralocaspica* is a C4 type halophyte with dimorphic seeds, whose difference in germination and dormancy has been well documented (Li *et al*. 2007; Wang *et al*. 2008; He *et al*. 2013). However, the effects of the dimorphic seeds on the descendants have not been well understood. Wang *et al*. (2014) recently compared the biomass and mineral profiles of plants from dimorphic seeds of *S. aralocaspica* under different levels of nutrient and salinity. The present work systematically investigated responses of growth, physiology and relevant gene expression of plants from different seed morphs to long-term salt treatment for the first time. Results indicate that plants grown from brown and black seeds showed no significant difference for most indexes, which suggests that the different properties between the dimorphic seeds of *S. aralocaspica* are not transferred to the descendants on growth and physiological responses with or without salinity and both types of plants require salinity in growth to cope with the saline habitat.

In the present study, plant growth of *S. aralocaspica* was stimulated by the increasing salt concentration, and no significant difference was presented between both types of plants. Similar results were observed from the seed dimorphic halophyte *S. splendens*: seedlings grown from dimorphic seeds all grew well at high salinity (400 mM) (Redondo-Gómez *et al*. 2008). Although the effect of salinity on growth varies among halophytes, many halophytes show optimal growth in concentrations ∼200 mM NaCl (Flowers *et al*. 1986), and organic dry mass is stimulated by growth in saline conditions in at least some dicotyledonous halophytes (Yeo and Flowers 1980; Glenn and O'Leary 1984). Many natural halophytes can grow at similar rates to glycophytes even though they use energy from the ion accumulation and compartmentalization for osmotic adjustment (Yeo 1983). Our results suggest that *S. aralocaspica* is a euhalophyte and can benefit from salt for vegetative growth.

In the present study, only the higher salt concentration (500 mM) caused the Na^+^ to accumulate to a significant level in plants of both seed morphs, whereas K^+^ concentration and the consequent K^+^/Na^+^ ratio were reduced, which indicates that competitive inhibition between Na^+^ and K^+^ absorption results in a change in the balance of intracellular K^+^–Na^+^. Under salinity, the up-regulation of the vacuolar H^+^-ATPase genes (*VHA*), *SsVHA-H* and *SsVHA-B* and the increase in the activity of V-H^+^-ATPase provide the proton-driving force for sequestering Na^+^ in leaf vacuoles of *Suaeda salsa* (Li *et al*. 2004, 2006). In the present study, the expression levels of B subunit of *V-H^+^-ATPase* gene were significantly increased under salt treatment in plants from black seed (*V-H^+^-ATPase*) and the expression of *V-H^+^-PPase* increased in plants from both seed types, but why the expression pattern of *SaV-ATPase* showed no obvious change in plants from brown seed was not clear. These results are in agreement with those found in another halophyte *Salicornia europaea*, in which the transcript accumulation of *SeVHA-A* and *SeVP1* (encoding for V-H^+^-PPase) in shoots increased significantly with rising NaCl concentration (Lv *et al*. 2012). This sequestration requires Na^+^ being actively transported into the vacuole against the electrochemical gradient and Na^+^ in the vacuole being prevented from leaking back into the cytosol (Bonales-Alatorre *et al*. 2013), the high expression of these genes and corresponding proteins play important roles in this process.

Proline, GB and SSs serve as the important organic osmolytes that contribute to alleviate the low cellular water potential (Sakamoto and Murata 2002; Flowers and Colmer 2008; Trovato *et al*. 2008). In the present study, with the increasing salt concentration, proline, GB and all SSs measured were significantly increased in plants from both seed morphs. Consistent with the above results, the expression levels of *P5CS* (for proline synthesis) and *BADH* (for GB synthesis) genes increased with the rising NaCl concentration in plants from both seed types. *SsP5CS* in *S. salsa* was also up-regulated under salt stress (Wang *et al*. 2002). The BADH level increased under salt treatment in *S. aralocaspica* (Park *et al*. 2009). The above results suggest that these osmolytes must have important function in osmo-regulation of the cells in *S. aralocaspica*.

Salt stress can generate secondary oxidative stress by production of excess reactive oxygen species (ROS), e.g. H_2_O_2_ and ${{\text{O}}_{2}}^{-}$ (Hernández *et al*. 2001; Xiong and Zhu 2002). In the present study, the production of ${{\text{O}}_{2}}^{-}$ and the concentration of H_2_O_2_ increased significantly in both types of plants with rising salinity. However, the concentration of the biomarker for the lipid peroxidation-MDA was significantly increased only at higher rather than lower salt concentration in both types of plants. These data indicated that only higher salinity may lead to damage of the cellular membrane (corresponding to significant accumulation of MDA), and the results suggest that the antioxidant agents must function actively to scavenge excess ROS under higher salinity.

The metabolism of ROS depends on the synergetic function of multiple antioxidant enzymes, in which SOD has been regarded as the first defence line by catalysing the dismutation of ${{\text{O}}_{2}}^{-}$ to molecular oxygen and H_2_O_2_ (Badawi *et al*. 2004; Ashraf 2009), and the H_2_O_2_ can be further scavenged predominantly by CAT and POD (Willekens *et al*. 1997; Azevedo Neto *et al*. 2006). In the present study, the activity of SOD significantly increased with the rising ${{\text{O}}_{2}}^{-},$ while that of CAT and POD decreased significantly in both types of plants under higher salinity, suggesting that the failure of an increase of the activity of CAT and POD might mean that the excess H_2_O_2_ was not effectively scavenged, thereby causing more serious oxidative stress. Several other enzymes also play important roles in detoxification of H_2_O_2_ via the AsA–GSH cycle (Bowler *et al*. 1992). Ascorbate peroxidase and GR, which are the first and last enzymes in this cycle, respectively, are responsible for H_2_O_2_ scavenging in green leaves (Foyer and Harbinson 1994). Ascorbate peroxidase employs AsA as a specific electron donor to reduce H_2_O_2_ to H_2_O (Asada 1992), and GR has a central role in maintaining the reduced GSH pool during stress (Pastori *et al*. 2000; Alhdad *et al*. 2013). In the present study, the activities of APX and GR increased significantly with rising salt concentration in both types of plants, which was consistent with the increasing trend of two corresponding non-enzymatic antioxidants AsA and GSH. These two agents were reported as the most powerful water-soluble antioxidants and could minimize the oxidative damage either by directly scavenging dangerous ROS or via the AsA–GSH cycle (Foyer *et al*. 1997; Ashraf 2009). The fully oxidized AsA has a short half-life and would be lost unless it is reduced back, in which GSH plays a key role in regenerating AsA via the AsA–GSH cycle (Foyer and Halliwell 1976; Gill and Tuteja 2010). GSH concentration usually declines with the increasing of stress intensity (Tausz *et al*. 2004). However, in the present study, the levels of AsA and GSH accumulated significantly when both types of plants were exposed to higher salt concentration, suggesting that these two non-enzymatic antioxidants play a predominant role in endowing this halophyte with resistance to potential oxidative damage compared with antioxidant enzymes.

Plant cells contain three SOD types (i.e. Fe-SODs, Mn-SODs and Cu/Zn-SODs) that differ in their metal cofactors (Attia *et al*. 2008). The transcriptional expression of the most abundant isoform, Cu/Zn-SOD (gene as *cSOD*), located in cytosol and chloroplast (Bowler *et al*. 1994), was analysed in the present study. Results showed that *cSOD* was up-regulated at low salt concentration, whereas down-regulated at higher salt concentration, which was incompletely correlated with the activity of SOD at higher NaCl concentration. This difference may correspond to post-translational regulation, inactivation of the enzymes by their product H_2_O_2_ or direct inhibition by Na^+^ at higher salinity (Scandalios 1993; Madamanchi *et al*. 1994). The transcriptional level of *SasAPX* in the present study increased significantly in both types of plants with the rising salt concentration, which was consistent with the corresponding enzyme activity: it suggests an important role of this gene in the protection against higher salinity-induced oxidative stress in *S. aralocaspica*. Analysis of the expression of *SaGR* in the present study showed that gene expression with the rising salt concentration correlated with the changes of GR activity and GSH concentration in *S. aralocaspica* plants from dimorphic seeds, which implies that the increases in gene expression and GR activity are not only required for the scavenging of ROS. Glutathione reductase activity needed sustained high levels of GSH for adjusting cellular redox homeostasis (Pastori *et al*. 2000; Contour-Ansel *et al*. 2006).

High salinity also considerably affects the process of photosynthesis in most plants by altering the ultrastructure of the organelles, concentration of various pigments and metabolites and enzyme activities involved in this process (Ashraf and Harris 2013). In the present study, the total Chl concentration significantly decreased after exposing plants to higher salinity, which may result in the destruction of the chloroplast structure. The impaired biosynthesis or accelerated degradation of photosynthetic pigment was caused by cholorophyllase (Rao and Rao 1981; Singh and Dubey 1995). In the present study, the activities of two key photosynthetic enzymes were measured and the results showed that activity of RUBPC was significantly decreased with the rising salt concentration, while that of PEPC increased significantly. It is known that *S. aralocaspica* is a kind of single-cell C4 plant species (Voznesenskaya *et al*. 2001), although RUBPC as major carbon fixation enzyme is the limiting factor of the rate of photosynthesis (Mitra and Baldwin 2008). In C4 species, CO_2_ is initially fixed by PEPC in the mesophyll cells (Aldous *et al*. 2014) and refixed by RUBPC (Sage 2004). Increased salt concentration is suggested to repress RUBPC activity of both glycophytes and halophytes (Osmond and Greenway 1972; Kaiser and Heber 1981). The decreased activity of RUBPC may be caused by a salt-induced reduction of total water content (TWC) and accumulation of proline (Sivakumar *et al*. 1998; Lawlor and Cornie 2002), which is in agreement with the results of TWC and proline analysis in the present study. The suppression of RUBPC activity by accumulated proline might weaken hydrophobic interactions between subunits of the enzyme and finally dissociating the small subunits from the large subunits (Sivakumar *et al*. 2001). However, the activity of PEPC can be used as biochemical indicator of salt tolerance (Guerrier 1988). The increased PEPC activity upon salt stress could potentially improve carbon metabolism during periods of reduced stomatal conductance by reassimilating respired CO_2_ and/or increasing rates of CO_2_ fixation at night when stomata are open (Cushman and Borland 2002; García-Mauriño *et al*. 2003; Carmo-Silva *et al*. 2008). In a C4 shrub *Atriplex lentiformis* (grown in saline habitats), the net CO_2_ assimilation rate and the PEPC activity in leaf increase linearly with salinity rising (Zhu and Meinzer 1999). Phosphoenolpyruvate carboxylase may also support the biosynthesis of biocompatible osmolytes such as proline (Chen *et al*. 2010). Being consistent with the corresponding enzyme activity, our data showed that the transcriptional level of *SaPEPC* was increased significantly, while that of *SaRUBPC* was decreased significantly in both types of plants with the rising salt concentration. An up-regulated *PEPC* in response to salinity or drought stress has been well documented in C3, C4 and crassulacean acid metabolism plants (Li and Chollet 1994; González *et al*. 2003; Carmo-Silva *et al*. 2008). Specifically, a major up-regulation of *PEPC* and concomitant down-regulation of *RUBPC* are typical of this response (Fontaine *et al*. 2003; Dizengremel *et al*. 2009).

There is evidence that brown seeds are able to germinate faster and at higher percentage than black seeds in species with dimorphic seeds (Li *et al*. 2005; Redondo-Gómez *et al*. 2008; Song *et al*. 2008; Wang *et al*. 2008). In the halophyte *Suaeda maritima*, large seeds have larger cotyledons than small seeds, which imply that more nutrient reserves can be used for better germination of the large *S. maritima* seeds (Wetson *et al*. 2008). In the halophyte *Atriplex triangularis*, large seeds produce larger plants than do in small seeds and this dichotomy can be maintained throughout the whole life of the species (Ellison 1987). Similarly, in *Hedypnois cretica* and *Crepis aspera*, larger peripheral achenes produced bigger seedlings than did in smaller central achenes and the effect of different seed size on plant growth could sustain up to 40 days post emergence in the progeny of both species (El-Keblawy 2003). We found in our previous observation that the seedlings from brown seeds of *S. aralocaspica* emerged earlier and with higher uniformity than those of black seeds; however, the difference between two types of seedlings became indistinguishable in 2–3 weeks. After stratification of black seeds, the seedlings from brown and black seeds showed similar performance in emergence time, uniformity and the size. Further comparison between plants derived from heteromorphic seeds in the present study, we found that there was no significant difference on the performance with most tested physiological parameters and relevant gene expressions with or without salinity (Table 3), most of the indexes were induced under salt treatment and most of the performance of physiological parameters was matched with corresponding gene expression patterns. It suggests that the difference between two types of seedlings in the early stage in *S. aralocaspica* arose because of different rates of emergence time rather than for other reasons.

**Table 3.** Summary of physiological and gene expression responses of plants derived from heteromorphic seeds of *S. aralocaspica* under long-term salt stress. +, increase; ++, significant increase; −, decrease; =, unchange; GB, glycine betaine; SSs, soluble sugars; GSH, glutathione; ASA, ascorbic acid; SOD, superoxide dismutase; POD, peroxidase; CAT, catalase; APX, ascorbate peroxidase; GR, glutathione reductase; Chl, chlorophyll; PEPC, phosphoenolpyruvate carboxylase; RUBPC, ribulose-1,5-bisphosphate carboxylase/oxygenase.

Type	Response	Osmolyte					Antioxidant		Antioxidative enzyme					Photosynthesis parameter		
		K^+^	Na^+^	Proline	GB	SSs	GSH	ASA	SOD	POD	CAT	APX	GR	Chl	PEPC	RUBPC
Plant from brown seed	Physiology	−	++	++	++	++	++	++	++	−	−	++	++	−	++	−
	Gene expression		=	++	++				++		++	++	++		++	−
Plant from black seed	Physiology	−	++	++	++	++	++	++	++	−	−	++	++	−	++	−
	Gene expression		++	+	++				++		++	++	++		++	−

## Conclusions

Our data suggest that different concentrations of NaCl stimulated similar active responses of all descendants from dimorphic seeds of *S. aralocaspica* on osmotic, antioxidative and photosynthetic systems, which were consistent with plant growth and morphological changes, as well as gene expression patterns (Table 3). Plants grown from both seed morphs all performed well at higher salinity, which may be attributed to synergetic actions among osmotic homeostasis, antioxidative defence and photosynthetic functions. Osmolytes (proline and betaine) were significantly increased and the excess ROS produced by higher salinity were scavenged by increased levels of antioxidant enzymes (SOD, APX and GR) and corresponding antioxidants (AsA and GSH). Enhancement of PEPC activity at high salt intensity should be a positive effect on maintaining normal photosynthesis. Most tested parameters showed no difference between plants from the two types of seeds, but a few were different: the significance of these few differences is not clear. Our results suggest that there was no carry-over of seed heteromorphism to adult plants in *S. aralocaspica* and plants grown from both types of seeds all benefited from salinity.

## Sources of Funding

This work was supported by the National Natural Science Foundation of China (31060027; 31260037; 31460043), Project for Training Young Talents of Xinjiang Uygur Autonomous Region (2013721013) and the open funding of the Xinjiang Key Laboratory of Biological Resources and Genetic Engineering (XJDX0201-2011-03).

## Contribution by the Authors

J.C. and X.Y.L. carried out 2 years experimental work and L.C. and J.J.X. joined in 1 year. H.Y.L. and J.C. wrote the manuscript. All authors contributed to experimental design, data analysis and commented on the manuscript.

## Conflict of Interest Statement

None declared.
